# The shape of the global causes of death

**DOI:** 10.1186/1476-072X-6-48

**Published:** 2007-10-23

**Authors:** Anna Barford, Danny Dorling

**Affiliations:** 1 Social and Spatial Inequalities Group, Department of Geography, University of Sheffield, Sheffield, UK

## Abstract

**Background:**

World maps can provide an instant visual overview of the distribution of diseases and deaths.

**Results:**

There is a particular geography to each type of death: in some places many thousands of deaths are caused by a particular condition, whilst other equally populous areas have few to no deaths from the same cause.

**Conclusion:**

Physicians and other health professionals often specialise in the specifics of causes, symptoms and effects. For some practitioners gaining a worldview of disease burden complements smaller scale medical knowledge of where and how people are affected by each condition. Maps can make health related information much more accessible to planners and the general public than can tables, text, or even graphs. Ten cartograms based on World Health Organisation Burden of Disease data are introduced here; alongside seven based on data from other sources. The Burden of Disease cartograms are the latest in a much larger collection of social, economic and health world maps.

## Introduction

In this paper we introduce a new collection of cartograms, depicting geographies of medicine, health care, disease and death. Cartograms have a long but sparse history in medical mapping. The history we can reconstruct is one of similar ideas being repeatedly rediscovered in both Britain and the United States, often with little knowledge of earlier discoveries. There are almost certainly examples of their discovery and use for medical mapping in other countries, given the spontaneity with which the idea appears to be independently reborn time and again (but we have failed to find them).

To our knowledge medical cartograms were first employed by Wallace in 1926 [1] to create a new base map of the counties of the State of Iowa that was explicitly designed to allow coloured pins to be placed on the map each representing a reportable disease notification. Any clustering of the pins on that map would be much more likely to represent an actual cluster of significance on the ground. Three decades later, in 1955, Ian Taylor [2] independently produced "an epidemiological map" for use in the British ministry of health upon which were drawn the then current boroughs of London, each sized as a box of equal height drawn with width in proportion to population and filled with crosses to represent the notifications of poliomyelitis in each borough during the year 1947. The height of the crosses within each box was thus proportional to the rate of notifications.

Fifteen years later (1970) in the second edition of his Atlas of disease mortality Melvyn Howe [3] employed a population cartogram of the United Kingdom upon which various squares and diamonds were placed representing the peoples of the major cities, towns, counties and boroughs. These were then coloured by the age and sex standardized rates of particular diseases as they effected the populations. In 1965, which was five years earlier, and back over in the United States Levison and Haddon [4] had demonstrated how it was possible to use a population by area cartogram of upstate New York to investigate whether spatial clustering of Wilm's tumour or cervical carcinoma was occurring there in the early 1960s. Back in Britain, Hunter and Young in 1971 [5] showed how cartograms could be used to plot the influenza epidemic of that decade across England and Wales using a series of cartograms.

The first use of computer-created population cartograms in medical mapping was pioneered in America by Selvin and his colleagues during the mid eighties [6-9], but still the benefits of using cartograms in medical mapping were not widely recognised, mainly because of the difficulties of creating cartograms by computer and their arbitrary nature. A series of cartograms of mortality rates by area for many different diseases appeared in a cartogram based atlas of Britain [10]. The new algorithm to produce cartograms suitable (in general) to use as a base for medical mapping was only developed and made available three or four years ago [11].

**Figure 1:**
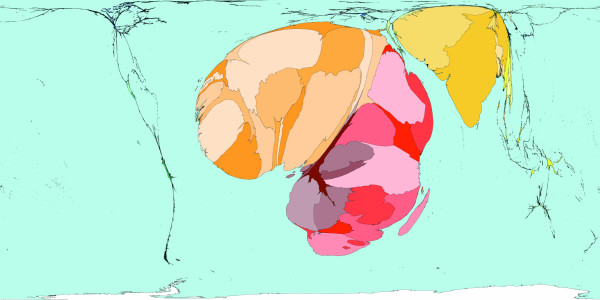
Worldmapper Map 413: Vitamin A deficiency deaths in 2002.

The new form of mapping which has recently been applied to world data, including mortality data [12-14], makes maps using an algorithm based on the physics of heat transfer [15]. This algorithm allows the density of a variable to become equal everywhere on the map. For example, on a map of deaths attributed to Vitamin A deficiency, the relatively large area of Pakistan denotes the relatively large proportion of all such deaths in the world that occur there (Figure 1). Similarly, Brazil cannot be seen on this map because very few Vitamin A deficiency-related deaths are thought to occur there. This scaling of the area of each territory by the number of deaths there due to a particular cause is achieved whilst allowing coastlines and borders to expand, contract and crumple. Thus territories appear distorted yet recognisable, somewhat like a caricature of the world. Note too that here we do not consider geographical variations within territory boundaries. For illustrations of sub-national variations in a range of measures see the Gapminder website [16].

Data availability is crucial to making these maps. Collecting and estimating good quality data that is internationally comparable is a challenging task. Recently the quality of World Health Organisation statistics has improved, particularly in terms of data accuracy and the number of territories for which data are collected and estimated. It is important of course to remember that the quality of data will vary both between territories and between causes of death, and that even the world total number of people dying each year is an estimate that is hard to verify. The original data, which is publicly available, includes a level of uncertainty indicator for each country for each cause and also a set of confidence limits for all cause mortality in each country. There are still issues of uncertainty even in that which is most certain. However, the situation now is far better than it was a decade ago. One example of dramatically improved data used here is the 'World Mortality in 2000: Life Tables for 191 Countries' [17]. This report was released because reporting of mortality data has been poor in many territories, despite the importance of this information for health policy.

The maps shown here are a subset of a broader mapping project, Worldmapper. These and many other health-related maps are available on the Internet and can be downloaded at no cost to the user. At the time of writing the Worldmapper project's health related maps include reshaping the world according to: health care quality, numbers of nurses working, physicians working, HIV prevalence, maternal mortality, stillbirths, infant mortality, malnutrition, malaria cases, hospital beds and spending on public and private health care. Other mapped indicators, which are also related to health, include maps of money, war, trade, labour, education and transport. Also available are the data used to make these maps, technical notes about the data used for these maps, and posters of these maps for use in education [18].

**Figure 2:**
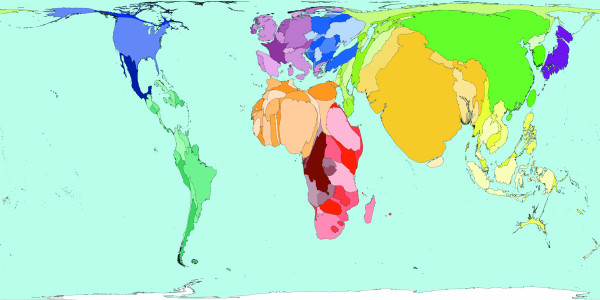
Worldmapper Map 368: All deaths (numbers in the year 2002).

**Figure 3:**
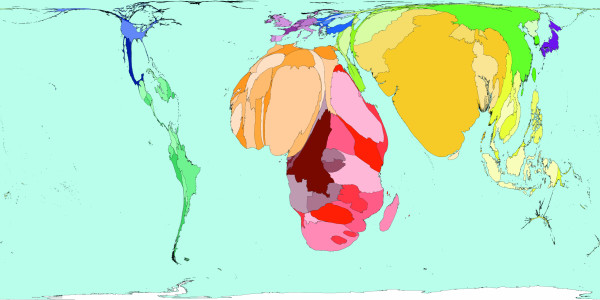
Worldmapper Map 371: Deaths from communicable, maternal, perinatal and nutritional conditions in 2002.

**Figure 4:**
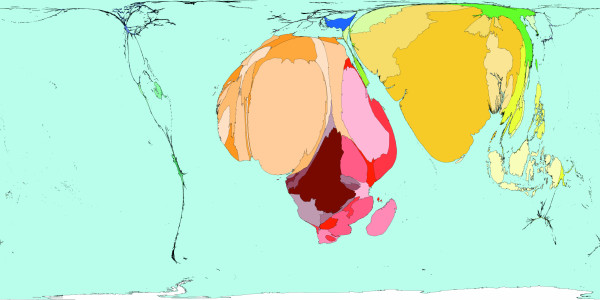
Worldmapper Map 380: Childhood cluster deaths in 2002 (includes: Pertussis, Poliomyelitis, Diphtheria, Measles and Tetanus).

**Figure 5:**
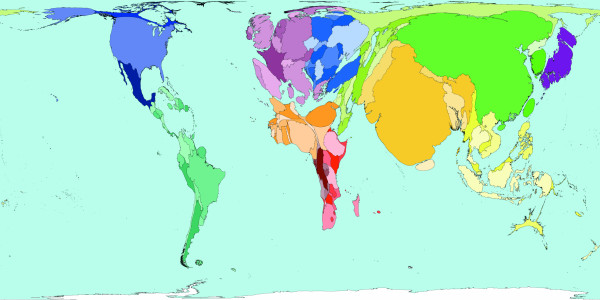
Worldmapper Map 417: All Chronic disease deaths in the year 2002.

**Figure 6:**
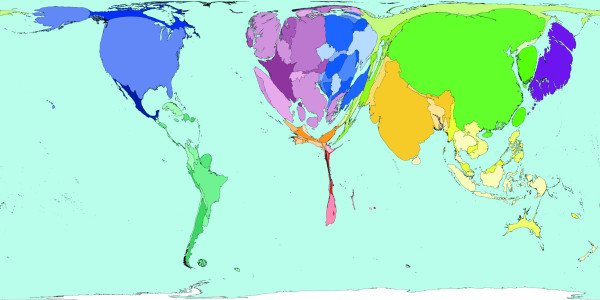
Worldmapper Map 425: Trachea, Bronchus and Lung Cancer deaths in 2002.

**Figure 7:**
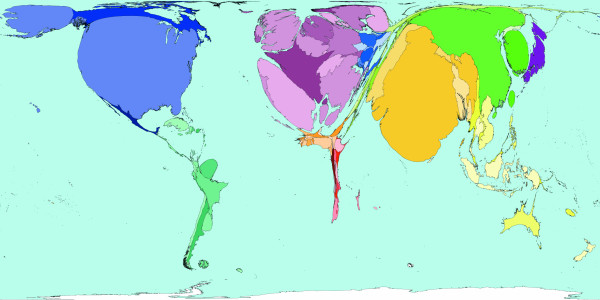
Worldmapper Map 444: Alzheimer and other dementias deaths in 2002.

**Figure 8:**
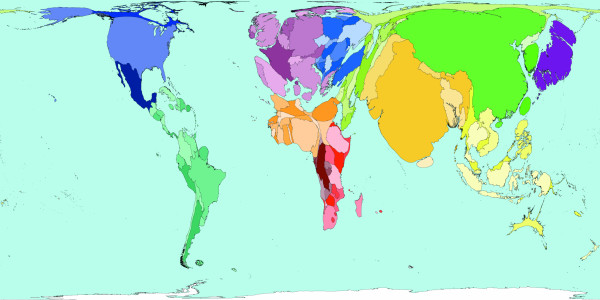
Worldmapper Map 367: Expected deaths (estimated for 2001).

Some 200 maps of disease and death are to be made available during 2007. A subset of these maps is shown below. Figures 1, 2, 3, 4, 5, 6, 7 and 8 rely on a related key source of data about death derived from the Global Burden of Disease Project [19], which provides world data on over 130 causes of death, ranging from Sexually Transmitted Infections, to Cancers, to Accidental Deaths. Figure 8 shows where deaths would occur if only age and sex determined age of death, and where you lived had no effect, it is based on the life table data [17] from the same source. The most recent references to these WHO projects now appear as book chapters [20] and journal articles [21].

**Figure 9:**
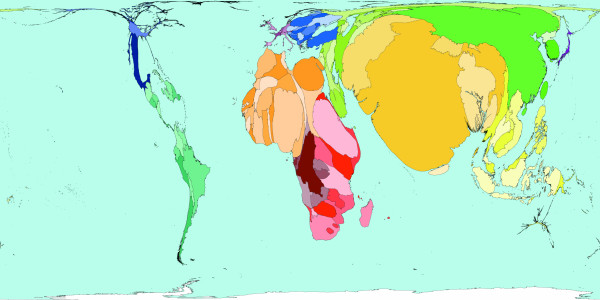
Worldmapper map 174: Human Poverty (as defined by the United Nations Development Programme).

In the following information about each of these maps, the Global Burden of Disease (GDB) number and International Classification of Disease (ICD) numbers are given for reference purposes. These maps have been selected to demonstrate the ways in which certain causes of death are distributed. This form of mapping may be one of the clearest ways to demonstrate the significance of the well-known axiom that poverty is linked to early death (Figure 9 shows the world distribution of poor people, measured by the United Nations Development Programme's Human Poverty Index). It is generally understood that in some places most young people die from preventable diseases, whilst elsewhere people survive long enough for the vast majority to die from the conditions of old age; but it is revealing to see the extent to which this is the case when the world is drawn to reflect the numbers.

**Figure 10:**
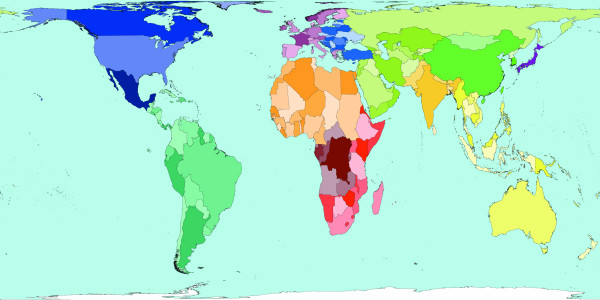
Worldmapper map 1: Land area. See [18] for further details.

**Figure 11:**
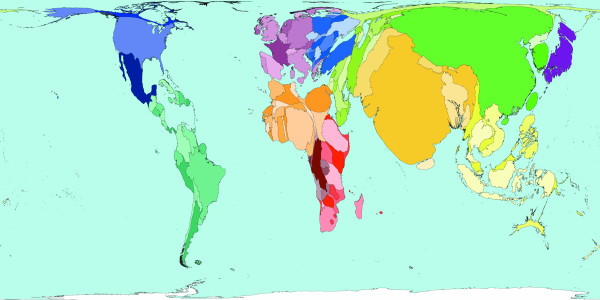
Worldmapper map 2: Total Population (estimated for 2002).

**Figure 12:**
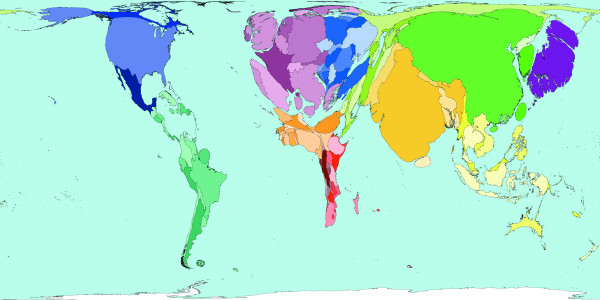
Worldmapper map 6: Total Elderly (people aged 65 years and older, estimated for 2002).

**Figure 13:**
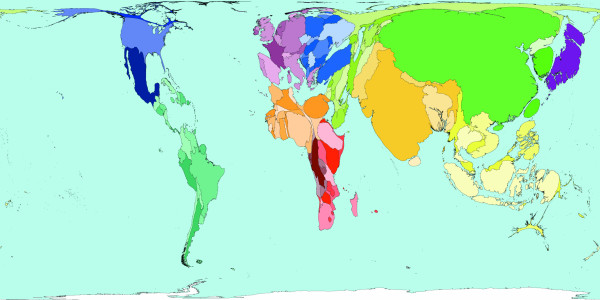
Worldmapper map 242: Men Smoking (numbers estimated for 2002).

To understand the distribution of diseases requires first understanding the global distribution of people. People are distributed very differently to land (the land distribution is shown in Figure 10). The distribution of people is the most simple map of who is at risk of disease and is shown in Figure 11 where areas are drawn in proportion to population in the year 2002. Of course, different people face different risks depending on a large number of factors. Figure 12 illustrates how these too are unevenly distributed by showing the world shaped by the elderly population aged 65 years or more. Figure 13 shows one additional risk factor for one group: the world shaped by the number of men who smoke. Lastly in introduction it is worth looking again at Figure 9 which shows the world shaped by the proportion of people living in poverty as internationally understood.

All the deaths estimated to have occurred worldwide in 2002 are mapped in Figure 2. That was a total of 57 million deaths. This map looks roughly similar to a map of population, although Latin America appears to have a lower proportion of all deaths occurring there, than the proportion of the world population that live there. India, China, Nigeria and the United States are where a large proportion of all deaths occurred in 2002 – these are also some of the more populous territories on earth. The original WHO source data groups diseases in a particular way and we have adopted their grouping here.

Roughly a third of all deaths in 2002 were caused by communicable, maternal, perinatal and nutritional conditions these can ostensibly almost all be controlled by public health interventions. The world shaped to show the locations of these 18 million deaths is drawn in Figure 3. The look of this map contrasts with the preceding map: territories in the South and East have generally expanded relative to those territories in the North and West. The anomalies are Australia and New Zealand, which have tiny areas due to both their small populations and the small number of deaths there due to these causes. [Source information: GDB cause U001 I. Communicable, maternal, perinatal and nutritional conditions; ICD9: 001–139, 243, 260–269,279.5, 280–281, 285.9, 320–323, 381–382, 460–465, 466, 480–487, 614–616, 630–676, 760–779, ICD10: A00-B99, G00-G04, N70-N73, J00-J06, J10-J18, J20-J22, H65-H66, O00-O99, P00-P96, E00-E02, E40-E46, E50, D50-D53, D64.9, E51-64].

The 1 million deaths used to shape Figure 4 are all those caused by vaccine-preventable childhood diseases. These diseases are Pertussis, Poliomyelitis, Diphtheria, Measles, and Tetanus. In many parts of the world these diseases do not threaten the lives of children – either because the disease has been locally wiped out, or vaccinations are used to protect almost all children, and/or treatment is available to those who do contract these diseases. This map shows that children living in South America, North America, Western Europe and Japan are usually quite safe from the threat of these diseases. Children living in Eastern Europe and the Middle East are also relatively unlikely to be killed by these diseases; it is in parts of Southern Asia and Africa where most children die due to childhood cluster diseases. It should be noted that where diseases do not result in death, serious disability may have been caused. [Source information: GBD cause U011 5. Childhood-cluster diseases, ICD 9: 032, 033, 037, 045, 055, 138, 771.3; ICD 10: A33-A37, A80, B05, B91]

Figure 1 shows a much rarer cause of death but a major cause of disability: Vitamin A deficiency (which is a major cause of blindness in the tropics). This map shows the distribution of the 20,000 deaths that this deficiency is estimated to have caused in 2002. The vast majority of these deaths occurred in African territories; most deaths outside of Africa were in India, Pakistan, Bangladesh, Nepal and Thailand. These deaths are related to education and the availability of vitamin A – a good and varied diet is enough to prevent almost all of these deaths. Vitamin A can be found in milk, carrots, green leafy vegetables and animals' livers. [Source information: GBD cause: U056, ICD 9: 264, ICD 10: E50].

Figure 5 represents the geography of all deaths caused by chronic diseases. Together with Figure 3, the deaths shown in this figure show the causes of all deaths attributable to disease worldwide. In 2002, there were 33.6 million deaths from chronic causes.  These deaths include cancers, diabetes, diseases of the heart, the respiratory tract and the digestive tract. This map looks similar to Figure 2; the most obvious exception is that a larger proportion of deaths from chronic diseases occur in Europe, whilst fewer occur in Africa. [Source information: GBD cause U059 II. Chronic diseases, ICD 9: 140–242, 244–259, 270–279 (minus 279.5), 282–285 (minus 285.9), 286–319, 324–380, 383–459, 470–478, 490–611, 617–629, 680–759; ICD 10: C00-C97, D00-D48, D55-D64 (minus D 64.9) D65-D89, E03-E07, E10-E16, E20-E34, E65-E88, F01-F99, G06-G98, H00-H61, H68-H93, I00-I99, J30-J98, K00-K92, N00-N64, N75-N98, L00-L98, M00-M99, Q00-Q99].

Cancers are a major cause of disease and each group of cancers can be used to draw a differently shaped world. The distribution of deaths shown in Figure 6 is of those due to cancers of the trachea, bronchus and lungs. These are all strongly smoking related. In 2002, 1.2 million deaths worldwide were due to these causes. These causes of death generally occur more in richer territories – the United States, Western and Eastern Europe, South Korea and Japan are prominent on this map. However, China also has a large area as more and more tobacco is sold there (historically mainly to men). Due to the low proportion of all people killed by the cancers in Africa, that continent has shrunk to become barely visible on this map. [Source information: GBD: U067 7. Trachea, bronchus and lung cancers; ICD 9: 162; ICD 10: C33-C34].

Figure 7 is a map of deaths from Alzheimer's disease and other dementias. These were thought to have caused and contributed to some 400,000 deaths worldwide in 2002. However, this diagnosis and the estimates based on it are especially error-prone. Many people do not reach an age where these conditions are likely, as they die from some other cause first. Other than India and China still being the location of high proportions of worldwide deaths (due to the large populations living in these territories), this map shows an inverse distribution to the map of deaths from childhood cluster diseases (Figure 4). India, China, Western Europe, the United States, Japan and South Korea have large areas on this map. [Source information: GBD cause U087 6. Alzheimer and other dementias; ICD 9: 290, 330, 331; ICD 10: F01, F03, G30-G31].

This short series of cartograms hopefully demonstrates quite clearly how where you live affects what you are likely to die from. It may also be of use to those already familiar with these statistics who have not seen them in this form before. The maps also allow us to experiment with alternative possibilities. Imagine if the world was changed, if from today onwards only our sex and age affected when we die; if where we lived became immaterial. Figure 8 shows the redistributed 57 million deaths that would be expected to have occurred, had worldwide average age-sex specific mortality rates applied everywhere. The statistics behind this figure have been calculated on the assumption that access to health care, prevalence of infectious diseases, and many other factors become equal. This map shows what could happen, Figure 2 shows what does happen.

The differences between Figure 2 and 8 are due chiefly to the differences in age composition (and less so gender). On Figure 8 the excess death count in the African continent shrinks considerably (because the populations in the countries in this continent typically have a much younger age structure), whereas the death count increases notably for the United States, Canada, and Europe (which have an older age structure), somewhat increases for Japan and China, and stays about the same for India.

**Figure 14:**
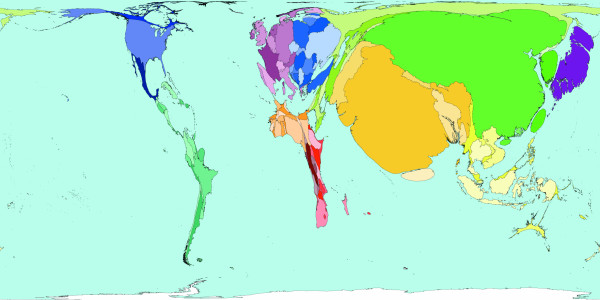
Worldmapper map 292: Self-inflicted deaths (estimated for 2002).

**Figure 15:**
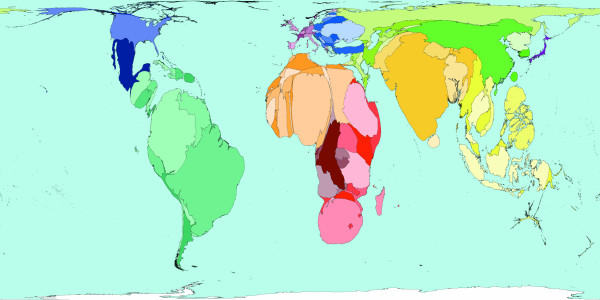
Worldmapper map 291: Violent deaths (estimated for 2002).

**Figure 16:**
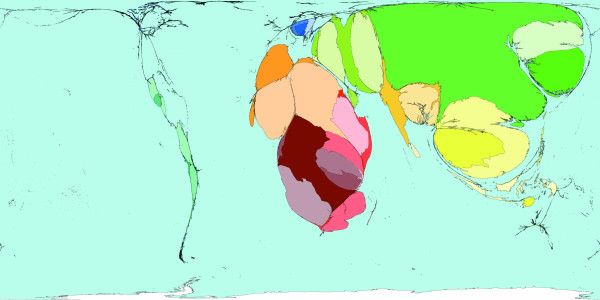
Worldmapper map 287: War Deaths 1945–2000.

**Figure 17:**
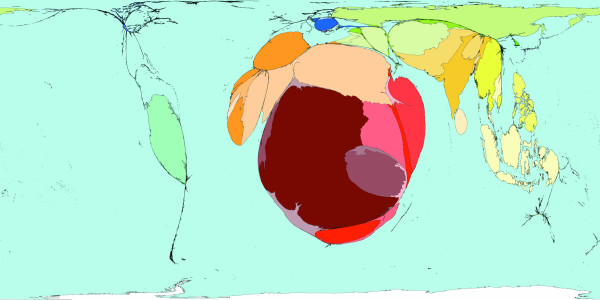
Worldmapper map 288: War Deaths 2002.

Policy makers could well benefit from seeing the world through images such as these. In some cases policy makers can have a far more immediate effect than others. There is evidence that government influences suicide rates [22]. Homicide rates also vary dramatically between nations and, most obviously, policy makers are the makers of war. Consider the worldwide distributions of suicides, homicides, war deaths from 1945–2000, and war deaths in 2002 (Figures 14, 15, 16 and 17). With colleagues we have tried to ensure that these images appear in a wide variety of media; they will have greater weight if they are familiar forms. This is particularly the case if they can be instrumental in the formation of public opinion and can effect what we request from our politicians. Examples of these maps appearing in the printed press are available on the Internet [23]. One way in which images such as this may come to be used more widely is if they are employed in teaching. To help in this we have provided all the data our maps are based on as freely downloaded spreadsheets from the website and many lecturers are currently using these resources in schools and universities.
